# Preparation of Sol-Gel Derived Anticorrosive Coating on Q235 Carbon Steel Substrate with Long-Term Corrosion Prevention Durability

**DOI:** 10.3390/ma12121960

**Published:** 2019-06-18

**Authors:** Yue Li, Chunchun Wu, Ming Xue, Jiawen Cai, Yi Huang, Hui Yang

**Affiliations:** 1 Zhejiang California International Nanosystems Institute, Zhejiang University, Hangzhou 310058, China; liyue8@zju.edu.cn (Y.L.); wuchun@zju.edu.cn (C.W.); whf5560@zju.edu.cn (M.X.); 2 Research Institute of Zhejiang University-Taizhou, Taizhou 318000, China; 3 School of Materials Science and Engineering, Zhejiang University, Hangzhou 310027, China; 21726110@zju.edu.cn (J.C.); 11826012@zju.edu.cn (Y.H.)

**Keywords:** sol-gel coating, corrosion prevention, Q235 carbon steel, filler, EIS

## Abstract

Anticorrosive coatings prepared by sol-gel derived approaches have become an emergent research area in the field of corrosion prevention materials. Furthermore, enhanced coating thickness can greatly improve the barrier effect of the sol-gel coatings, thus influencing their service life in industrial applications. Here, we propose the preparation of a two-layer coating system using a low-cost sol-gel derived method. The coating structure was composed of first an underlying layer incorporated with silica and titania powder as filler and pigment materials, and a second translucent topcoat containing a colloidal silica sol-gel matrix crosslinked by methyltrimethoxysilane (MTMS). This coating system was applied on Q235 carbon steel substrate by a two-step spray deposition method, resulting in an enhanced coating thickness of around 35 μm. The physical and morphological properties of the coatings were characterized using multiple techniques, including scanning electron microscopy (SEM), energy dispersive spectroscopy (EDS) and atomic force microscopy (AFM). The anticorrosion performance of the sol-gel coatings was studied by a salt spray test, outdoor exposure test and electrochemical impedance spectroscopy (EIS). Results revealed that this two-layer coating system exhibited excellent physical and anticorrosion properties, and that the topcoat played a crucial role in maintaining the barrier effect and preventing water leakage.

## 1. Introduction

Corrosion of commonly used metals, such as carbon steel, stainless steel, and aluminum alloy, poses a major threat to various applications ranging from infrastructure to machinery. It is believed that corrosion accounts for the economic loss of 2–5% gross domestic product globally [1]. Among these metals, Q235 carbon steel is one of the most widely used in industry owing to its moderate carbon content, good mechanical performance and excellent welding properties. However, carbon steel is notoriously known for its susceptibility to corrosion in many environments. To improve the corrosion resistance of metals, metallic surfaces are mostly protected by anticorrosive films or coatings that function as a physical barrier between corrosive media and substrates to prevent corrosive species from reaching the metallic surfaces, or act as inhibition species to inhibit the corrosion process [2]. Traditional chromate-based chemical conversion coatings (CCCs) are effective corrosion prevention materials for a variety of metals. Compared to other corrosion inhibitors, the high oxidation properties of the hexavalent chromium-containing compounds make them more effective in preventing corrosion development [3]. The pre-treatment of metallic surfaces with chromium-containing compounds also enhances the adhesion strength between the coating and metallic surface. However, CCCs are increasingly regulated or restricted in many countries over the past decades due to their high toxicity to humans and the environment [1]. In addition, many disadvantages associated with traditional coating systems, such as fast thermal aging, low thermal resistance, and poor mechanical properties, result in short service life and limit their applications in harsh environments.

In recent years, rapid developments have been made in the area of ceramic-based anticorrosive coatings prepared using sol-gel derived approaches [4,5,6,7,8,9,10,11,12,13,14,15,16], and they are considered to be one of the most promising substitute materials for traditional chromate pre-treated anticorrosive coatings. The versatility of sol-gel approaches enables tunable chemical and mechanical properties of the protective coatings, and consequently brings about various functionalities of sol-gel based coating systems. Moreover, the hydrolysis and condensation reactions of metal alkoxides in sol-gel processes can form strong chemical bonds with various metallic surfaces, enabling the creation of dense, uniform and defect-free protective films on metallic substrates. Silica-based coatings, in particular, have excellent thermal resistance and chemical stability arising from the high binding energy of Si–O covalent bonds (1014 kJ/mol) [17]. On the other hand, Si–O–M bonds can be formed between silicon hydroxyl groups and hydroxyl groups on metal surfaces, giving the coatings higher adhesion to the metallic surfaces [9,18].

As more advancements have been achieved in the field of sol-gel based coatings, many strategies involving the incorporation of filler materials into sol-gel coating structures have emerged in order to improve some of the coating properties. In this regard, various nano- or micro-sized filler materials have been used, including silica nanoparticles [19,20,21,22,23], graphene oxide [24], carbon nanotubes [25], nanocontainers [26,27], and halloysite nanotubes [28,29]. For example, Nezamdoust et al. prepared phenyl-trimethoxysilane sol-gel coatings incorporated with carbon nanotubes, and achieved enhanced corrosion resistance [25]. Other studies incorporated inhibitor-loaded nanocontainers into sol-gel matrices to enable the self-healing functionalities of the coating systems upon exposure to corrosive electrolytes [26,27]. Among these filler materials, silica nanoparticles have drawn increasing attention as many studies have shown that silica nanoparticles embedded in sol-gel coatings can increase the coating thickness, density and surface hardness, and ultimately enhance the barrier effect of sol-gel coatings [19,20,21,22,23]. Moreover, the design of sol-gel coatings with multiple layers can bring about various functionalities, such as active corrosion protection, high coating adhesion and thickness, high surface hydrophobicity, scratch resistance, and aesthetic properties [4,13,22,27,30]. For example, Tan et al. previously reported that sol-gel coatings consisting of multilayer structures could improve their anticorrosion properties on Mg surfaces through reduced levels of coating porosity [13]. Other studies have also demonstrated that the incorporation of anticorrosive inhibitors into different layers of multilayer sol-gel coatings could lead to varied self-healing functionalities [4,22].

In general, coating structures with a higher coating thickness and sealing quality usually lead to better anticorrosion performance [31]. Currently, the preparation of sol-gel based anticorrosive coatings with high thickness remains a great challenge, thus leading to unsatisfactory anticorrosive properties [2,18,30,32]. This is mainly due to the higher probability of crack and defect formation when thicker coating films are deposited, especially for the inorganic sol-gel coating systems [31,33]. In addition, most previous studies used solely silane precursors to constitute the majority of the coating materials. These synthesis methods often require the use of expensive organic silane raw materials, and in most cases are unable to create coatings with desirable thicknesses and colors that meet industrial standards and aesthetic requirements. The deposition techniques commonly used in these coating preparations were dip-coating or spin-coating [34,35,36], which usually require multiple cycles of deposition procedures, and the as-prepared coatings were often less than 20 μm in thickness [37,38,39]. In this regard, sol-gel derived coatings incorporated with nano- or micro-sized filler and pigment materials and prepared using spray deposition can be a solution for creation of coating systems with higher thicknesses, enhanced anticorrosion properties, and selectable color preference.

In the present work, we report a facile and simple method for the preparation of a sol-gel derived coating system, where a two-layer coating structure on Q235 carbon steel substrate was designed and prepared by layer-by-layer spray deposition of a reacted mixture of commercially available inorganic colloidal SiO_2_ nanoparticles and a commonly used silane crosslinker, methyltrimethoxysilane (MTMS). Silica and titania powder were selected as filler and pigment materials in this study due to their low-cost and good compatibility with other chemical components in this reaction system. Incorporation of these filler materials into the underlying layer of the coating structure resulted in protective films with increased coating thickness and white colored appearance. A translucent topcoat containing only crosslinked silica nanoparticles was then applied onto the underlying layer in order to improve the surface hydrophobicity and enhance the barrier properties. Several characterization techniques, including scanning electron microscopy (SEM), energy dispersive spectroscopy (EDS), and atomic force microscopy (AFM) were employed to examine the morphological and physical properties of the two-layer coating structure, as well as the underlying coating layer without the topcoat. The anticorrosion properties of the coating structures were then investigated by salt spray test, outdoor exposure test and electrochemical impedance spectroscopy (EIS), and the results indicated that the two-layer coating system with enhanced coating thickness exhibited good corrosion prevention durability. Comparative EIS studies also revealed that the application of the more uniform and hydrophobic topcoat played an important role in this coating system.

## 2. Materials and Methods

### 2.1. Materials

Colloidal SiO_2_ nanoparticles (A50, mean particle size ~50 nm, 50 wt.% in water) were purchased from Akzo Nobel Co. Ltd. (Taipei, Taiwan). Titanium dioxide (TiO_2_) powder (R902, average particle size ~0.405 μm, >93%) was supplied by DuPont Co. Ltd. (Shanghai, China). Silica whisker powder (particle size 1–5 μm) was purchased from Shanghai Huijingna New Material Co. Ltd. (Shanghai, China). Methyltrimethoxysilane (MTMS, 99%) was provided by Qufu Chenguang Chemical Co. Ltd. (Qufu, China). Hydrochloric acid (HCl, 36%) and acetic acid (HAc, AR) were purchased from Sino Pharm Chemical Reagent Co. Ltd. (Shanghai, China). 150 mm × 75 mm × 1 mm or 150 mm × 100 mm × 1 mm Q235 carbon steel panels were provided by Baoshan Iron & Steel Co., Ltd. (Shanghai, China).

### 2.2. Preparation of Sol-gel Coatings

#### 2.2.1. Preparation of Liquid Dispersion for Undercoat

To begin with, a stock slurry mixture was prepared by pre-dispersing 2120 g of colloidal SiO_2_ suspension, 670 g of silica whisker powder, and 500 g of TiO_2_ pigment powder in a high-speed dispersing machine (Biuged Laboratory Instruments (Guangzhou) Co. Ltd., Guangzhou, China, BGD750/10) at 500 RPM/min for 30 min. The obtained mixture was then milled for 1 h using a horizontal ball mill (Dongguan Longly Machinery Equipment Co. Ltd., Dongguan, China, NT-V6L). Subsequently, 324 g of the stock mixture was taken out, whose pH was then adjusted to 4.0 by adding ~5 mL of 5% HCl solution dropwise into the mixture. After the pH adjustment, 134 g of MTMS was added into the mixture to initiate the hydrolysis and condensation reactions, and the reactions were allowed to continue for 8 h at room temperature under constant agitation using a tube roller mixer working at 300 RPM/min.

#### 2.2.2. Preparation of Liquid Dispersion for Topcoat

200 g of colloidal SiO_2_ suspension and 40 mL of distilled water were first mixed by high-speed dispersing machine for 10 min, and the pH of mixture was then adjusted to 3.0 by adding 7.5 mL of HAc, followed by addition of 120 g of MTMS. The reaction was allowed for 7 h at room temperature under agitation using a tube roller mixer operating at 300 RPM/min.

#### 2.2.3. Coating Deposition

Before the deposition procedure, the as-received carbon steel panels were sand blasted by quartz sands (0.5 mm particle size), and washed and rinsed using an ethanol/distilled water mixture with ultrasonication. For the two-layer coating, the underlying layer was deposited by uniformly spraying the white mixture described in Section 2.2.1 on clean and dry carbon steel substrates using a pressurized spraying gun. The first coating layer was allowed to dry at room temperature for 5 min, followed by deposition of the second translucent top coating layer by the same process. The metal panels coated with sol-gel films were then cured at 170 °C for 30 min at a 5 °C·min^−1^ heating ramp, after which the specimens were allowed to cool down to room temperature in an ambient environment. For comparative study, one-layer coating samples were also prepared following the same deposition and curing procedures without application of the translucent topcoat.

### 2.3. Characterizations

The morphologies of the SiO_2_ nanoparticles and filler mixture before and after the sol-gel reactions were observed by a transmission electron microscope (TEM, JEM-1200EX, JEOL, Tokyo, Japan). The microstructure of the coating layers was investigated by a field-emission scanning electron microscope (SEM, 01-43, Zeiss Sigma, Cambridge, UK) at a working voltage of 15 kV. To obtain the cross-sectional SEM images of the coating layers and steel substrate, small metal chips covered with coating films were saw cut and polished. The elemental composition data was acquired on an energy dispersive spectrometer (EDS, Nano Xflash Detector 5010, Bruker, Billerica, MA, USA) mounted in the scanning electron microscope. The topological structure of the coating surfaces was scanned using an atomic force microscope (AFM, MultiMode, VEECO, Oyster Bay, NY, USA).

EIS measurements were performed using an electrochemical workstation (PARSTAT 4000A, Princeton Applied Research, Princeton, NJ, USA). Triplicate sol-gel coating samples with and without the topcoat on carbon steel substrates were prepared for the EIS measurements. A circular coating surface area with diameter of 4 cm was exposed to a 5 wt.% NaCl solution in a three-electrode plastic cylindrical cell, and platinum wire electrode and Ag/AgCl reference electrode were used in all tests. The spectra were acquired in the frequency range from 10^−2^ Hz to 10^5^ Hz with an amplitude of the sinusoidal voltage of 50 mV.

### 2.4. Coating Performance Testing

Optical photographs of the test samples were acquired using a commercial digital camera or a photo scanner. Pencil hardness was tested using a pencil hardness tester (BGD 506, Biuged Laboratory Instruments Co., Ltd., Guangzhou, China) following ASTM D3363 standard. Adhesion strength was tested using both cross-cut and pull-off methods. In the cross-cut test, a cross hatch adhesion tester (BGD 502 Biuged Laboratory Instruments Co., Ltd., Guangzhou, China) in accordance with ISO2409:2013 standard was used. The pull-off measurements were performed using a pull-off adhesion tester (Positest AT-M, Ogdensburg, NY, USA) following the ISO 4624:2016 standard. Static water contact angle measurements were conducted by dispensing droplets of deionized water on coating surfaces using a contact angle meter (OCA50AF, Data Physics, Filderstadt, Germany). Coating thickness was measured using a handheld coating thickness gauge (TT260, Beijing Zhongyi Technology Co., Ltd., Beijing, China), and the average thickness values and standard derivations were calculated based on measurements of at least 10 samples.

### 2.5. Salt Spray Testing

The neutral salt spray tests were carried out using a salt spray fog chamber (KD-90, Dongguan Kedi Instrument Industry Co., Ltd., Dongguan, China) in accordance with the ISO 9227:2017 standard. To protect the uncoated edges of the metal panels, the peripheries of the specimens were masked with a mixture of paraffin and rosin. Triplicate sol-gel coating protected carbon steel coupons were placed into the salt spray chamber at an angle of 15° and exposed to the continuous atomized spray of 5.0 wt.% NaCl solution at pH = 6.5–7.0 and T = 35 °C.

### 2.6. Outdoor Exposure Testing

Outdoor exposure tests were conducted following the ISO 8565:2011 standard at an outdoor facility located in Hangzhou, China, where typical subtropical monsoon climate characteristics are prevalent. Triplicate sol-gel coated carbon steel specimens with masked edges along with uncoated bare carbon steel coupons were securely mounted onto stainless steel sample racks equipped with ceramic separators, and the specimens remained untouched over the whole test period of 6 months from December 2018 to May 2019.

## 3. Results and Discussion

### 3.1. Preparation of the Sol-gel Coatings

**Figure 1:**
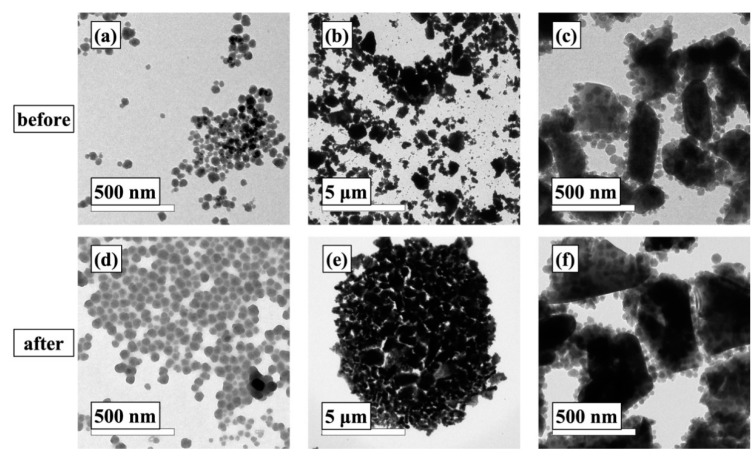
TEM images of colloidal silica nanoparticles (**a**,**d**) and silica nanoparticles/filler mixture before (**b**,**c)** and after (**e**,**f**) the sol-gel process.

Preparation of the one-layer and two-layer silica coatings was adapted from well-established sol-gel derived methods [18]. The sol-gel liquid mixtures used for coating deposition were mainly composed of colloidal silica and micro-sized filler particles crosslinked through reactions with MTMS during the sol-gel process. The TEM image shown in Figure 1a revealed that the commercially available colloidal silica used as the starting component for the sol-gel matrix formation was mainly comprised of spherical nanoparticles with an approximate size of 50 nm in diameter. The beginning material used for the undercoat was a particulate dispersion prepared by mechanically mixing the SiO_2_ nanoparticles, silica filler powder and titania pigment powder until a homogeneous white-colored mixture was formed, and the translucent mixture for the topcoat was prepared without addition of any filler materials. Figure 1b,c show that the SiO_2_ nanoparticles were uniformly situated on the surface of larger filler particles. The particulate mixtures then reacted with the MTMS crosslinker under acidic conditions via a hydrolysis and condensation reaction route. The TEM images of nanoparticles and fillers after 8 h of reaction are shown in TEM images in Figure 1d–f. It is clearly seen that, due to the interaction with MTMS, both SiO_2_ nanoparticles and silica/filler mixture agglomerated into larger crosslinked networks.

**Figure 2:**
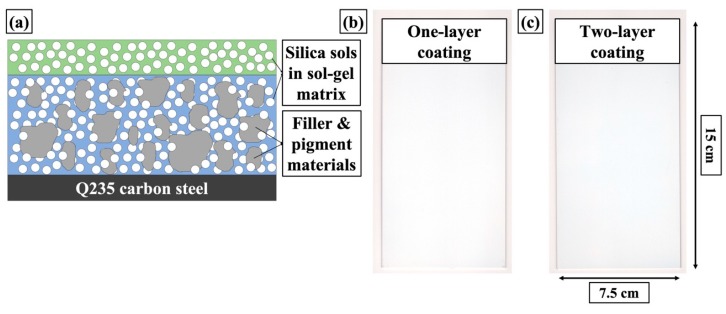
(**a**) Schematic demonstration of the two-layer coating structure; pictures of as-prepared coating surfaces with (**b**) one-layer and (**c**) two-layer structure.

**Figure 3:**
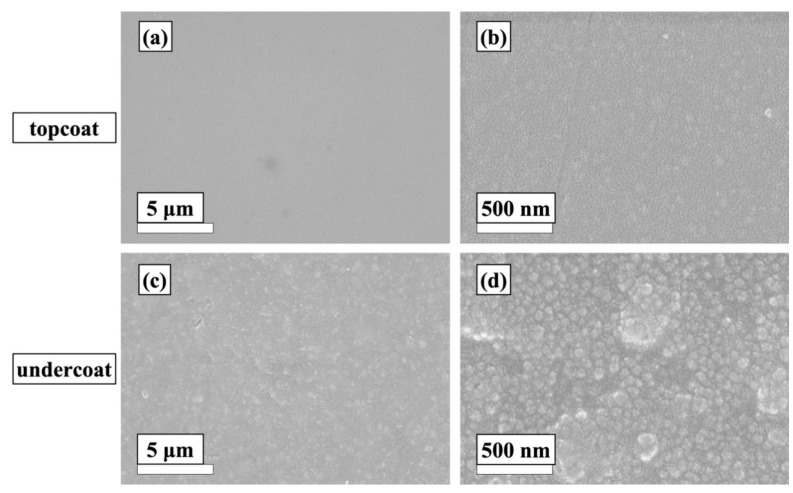
Low and high magnification top-view SEM images of (**a**,**b**) two-layer coating, and (**c**,**d**) one-layer coating.

The aged mixtures were subsequently deposited onto the sand blasted steel surface following a lay-by-lay deposition procedure to form a two-lay coating structure, as illustrated in the schematic in Figure 2a. Condensation polymerization of the coating films and crosslinking of the nanoparticles were undertaken under mild heat treatment. Figure 3b,c presents pictures of both one-layer and two-layer coatings on Q235 carbon steel substrates after the curing process, which showed uniform and homogenous coating surfaces without signs of cracks and grains. Compared to the one-layer coating structure without the translucent topcoat, the two-layer coating showed a slightly glossier surface. The coating thickness measured by coating thickness gauge revealed that the underlying layer and two-layer coatings were 27 ± 2 μm and 35 ± 3 μm in coating thickness, respectively. This unique two-layer coating structure contained a large portion of inexpensive silica and titania fillers that functioned as scaffolds in the coating structure and exhibited good compatibility within the sol-gel matrix, meanwhile significantly increasing the coating thickness compared to conventional silica sol-gel coatings [40].

### 3.2. Coating Morphological Characterization

#### 3.2.1. SEM Analysis

**Figure 4:**
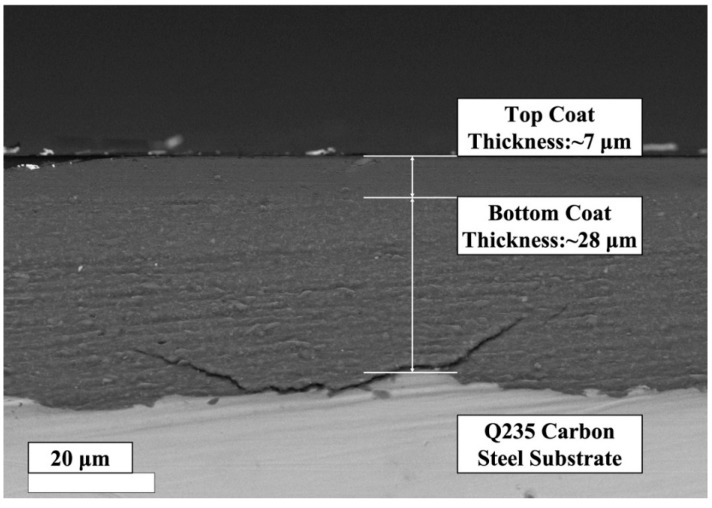
Cross-sectional SEM image of the two-layer coating structure.

The top-view SEM images of the two film surfaces (depicted in Figure 3) showed uniformly distributed surfaces without the presence of defects and micro-cracks. In the case of the two-layer structure (Figure 3a,b), the top surface exhibited smoother surface morphology that contained only closely packed SiO_2_ nanoparticles. In comparison, the underlying coating layer possessed more morphological features, including the presence of large filler materials that were randomly distributed on underlying film, leading to higher surface roughness. The cross-sectional SEM image presented in Figure 4 confirmed the successful formation of two-layer coating structure with a clear boundary between the two layers. The thicknesses of the topcoat and undercoat measured in SEM were around 7 μm and 28 μm, respectively, making the total coating thickness around 35 μm, which is in good agreement with coating thickness measured by the thickness tester.

#### 3.2.2. EDS Analysis

EDS analyses were conducted on different areas of the coating layers, and the data is summarized in Table 1. The signals collected from both top sections and cross sections of same coating layer presented similar elemental composition values. The results revealed the coatings were mainly composed of Si and O. In comparison with the top layer, the underlying structure showed an evident occurrence of Ti element, indicative of the successful incorporation of titania pigment powder into the coating structure.

#### 3.2.3. AFM Analysis

**Figure 5:**
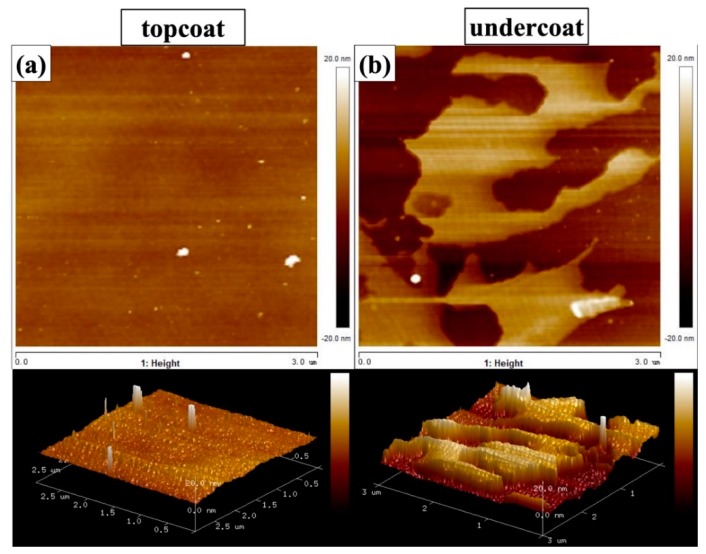
Atomic force microscopy (AFM) micrographs of top layer (**a**) and underlying layer (**b**) of the coating.

To confirm the surface topology and evaluate the surface roughness of the as-prepared coatings, AFM analysis was conducted on both layer surfaces. It can be seen from the 2D and 3D micrographs in Figure 5 that the surface topology of the underlying layer showed relatively rougher surface features in comparison with the top layer, consistent with the observations from the SEM analysis. The calculated root mean square (*R*_q_) surface roughness of filler-containing underlying layer was 5.86 nm, whereas the *R*_q_ surface roughness value of the top layer was 1.04 nm. The significantly larger surface roughness of underlying layer can be ascribed to the addition of silica and titania fillers. The declined surface roughness after the second layer deposition may imply that there is better corrosion resistance for the two-layer structure as opposed to the one-layer coatings without the pure silane top layer, since the rougher surface features may have provided more passages for corrosive media to reach the coating/metal interface and initiate corrosion reactions. A detailed description will be discussed in the EIS analysis.

### 3.3. Coating Anticorrosion Performances

#### 3.3.1. Physical Properties

The pencil hardness tests revealed that both coatings on carbon steel substrates showed a good pencil hardness higher than 9 H. Adhesion tests using the cross-cut test method demonstrated level 0 adhesion strength for both coating structures. Both the pencil hardness test and cross-cut adhesion test results showed the highest hardness and best adhesion levels for the corresponding test method. Moreover, the pull-off adhesion tests revealed adhesion strengths of 8.3 MPa for both coatings, which indicated tenacious adhesion at the coating/carbon steel interface and significantly higher adhesion strength compared to most of epoxy-based coatings on stainless steel or carbon steel [24,41]. In addition, no delamination between the topcoat and undercoat was observed, suggesting that the two-step deposition technique did not interfere with the covalent bonding formation and cause any interfacial debonding between the two layers.

**Figure 6:**
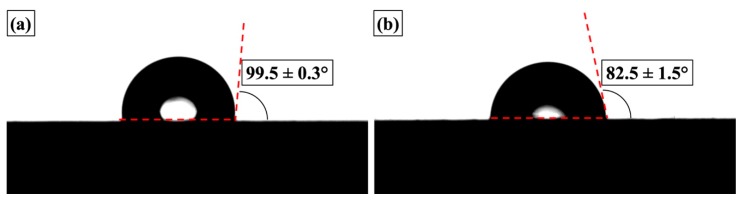
Contact angle measurements conducted on coating surfaces of (**a**) top layer and (**b**) underlying layer.

Water contact angle measurements were conducted to investigate the wettability and hydrophobicity properties of the coating surfaces. As shown in Figure 6, the contact angle of underlying coating surface was 82.5 ± 1.5°, and that of top coating surface increased to 99.5 ± 0.3°, indicative of the slightly hydrophobic nature of both surfaces. The increased hydrophobicity of the top layer surface mainly resulted from the increased content of hydrophobic methyl groups. On the other hand, the incorporation of filler and pigment materials in the silica sol-gel matrix resulted in increased exposure of hydroxyl groups to the surface of the underlying layer. These hydroxyl groups could not be fully hydrolyzed with MTMS, thus creating more surface charges and reducing the surface hydrophobicity.

#### 3.3.2. Salt Spray Test

**Figure 7:**
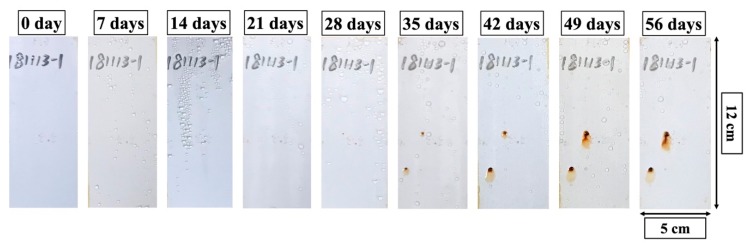
Pictures of two-layer coating specimen on Q235 carbon steel substrate after various days of the standard neutral salt spray test.

The pictures of the two-layer sol-gel coating after various days of neutral salt spray test are presented in Figure 7. No noticeable corrosion spots were observed for the first 28 days of the salt spray test. After 35 days, two evident pitting corrosion spots started to form, and enlarged corrosion product accumulation was observed in the following test period. By the end of the 56-day test period, the rest of coating surface remained intact without additional corrosion formation, or exfoliation of the coating materials from the steel substrates. Although most of the coating surface area was free of defects, there still existed some weak points from which corrosion reactions could initialize. These weak points might have been caused by the dust particles fallen on the coating surface during the spray deposition process.

#### 3.3.3. Outdoor Exposure Tests

**Figure 8:**
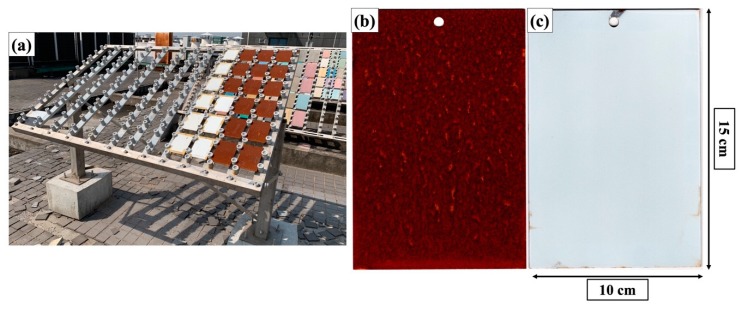
Optical photographs of (**a**) the coated and uncoated carbon steel coupons placed on the outdoor exposure sample rack during the exposure test, and (**b**) uncoated Q235 carbon steel coupon and (**c**) carbon steel coupon coated with the two-layer protective coating after 6 months of outdoor exposure test.

To test the anticorrosion performance of the coating system in real life scenarios, an outdoor exposure test was conducted. As shown in the picture in Figure 8a, the coating-protected Q235 steel coupons were placed at an outdoor test site located in Hangzhou, China for 6 months, a period of time during which the weather conditions were mostly rainy. Optical photographs of the retrieved samples protected by the coating showed no visible signs of corrosion (depicted in Figure 8c), whereas the bare steel coupons were heavily corroded and fully covered with rust (illustrated in Figure 8b). This comparison suggests that this coating system is effective in preventing the development of corrosion in Q235 carbon steel under such climate conditions. Due to the time constraints of this study, the full length of service life could not be fully realized. Longer exposure tests that span the whole service life of this coating system and studies on evolution of defects and corrosion developments will be conducted in future works. Taken together with the results from the salt spray tests, the effective metal protection and corrosion prevention capabilities of this coating system can be expected.

#### 3.3.4. EIS Measurements

The corrosion prevention performance of the sol-gel coatings was also investigated using EIS measurements. In this study, 5 wt.% NaCl solution was used for the immersion instead of the more commonly used 3.5 wt.% solution in order to accelerate the corrosion process. After different immersion times, the EIS spectra were collected in a frequency range from 10^−2^–10^5^ Hz over test periods of 99 days and 16 days for the two-layer coating and one-layer coating, respectively.

**Figure 9:**
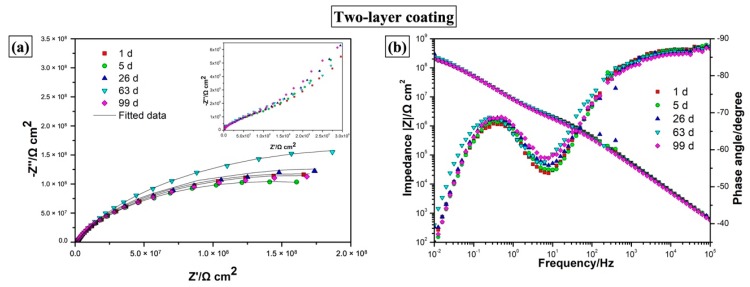
(**a**) Nyquist plots and (**b**) Bode plots obtained from electrochemical impedance spectroscopy (EIS) measurements for the two-layer coating immersed in 5% NaCl after different immersion times (1 d, 5 d, 26 d, 63 d, 99 d). The solid lines show the fitted experimental data.

**Figure 10:**
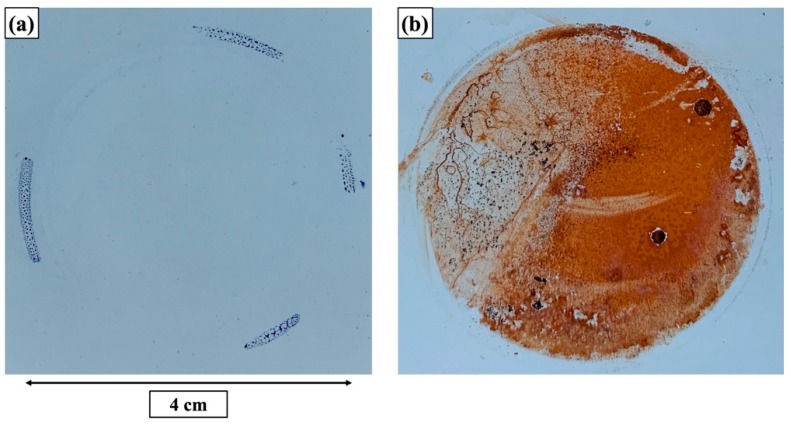
Optical images of (**a**) two-layer coating surface after 99 d immersion and (**b**) one-layer coating after 16 d immersion in 5 wt.% NaCl solution.

Nyquist plots and Bode plots for the two-layer coating structure presented in Figure 9 show good barrier properties of the coating structure, indicated by the capacitive impedance arcs with large radius in the Nyquist plots in Figure 9a and relatively high absolute impedance values (|Z|) in the Bode plots in Figure 9b. The low frequency impedance value |Z|_0.01Hz_ fell in the range of 10^8^–10^9^ Ω·cm^2^, which is relatively high compared to other coating systems. The phase angle spectra presented in Figure 9b also revealed two time constants positioning in the high-frequency range and low-frequency range, respectively. Here, the high-frequency time constant can be attributed to the top coating layer, and the low-frequency time constant can be assigned to the underlying layer. The low-frequency time constant for the two-layer coating structure is highly unlikely to correspond to the coating/metal interface since there is no evidence of the large mass transport of corrosion products between the metallic surface and corrosive electrolyte, indicated by the photograph of the two-layer coating surface (seen in Figure 10a) showing no signs of corrosion developments after 99 days of immersion in the EIS measurements. It is also worth noting that the Bode plots of the two-layer coating did not show significant decrease in impedance values with increased immersion time, indicative of the durable barrier effect and outstanding long-term anticorrosion performance of the multilayer coating structure.

**Figure 11:**
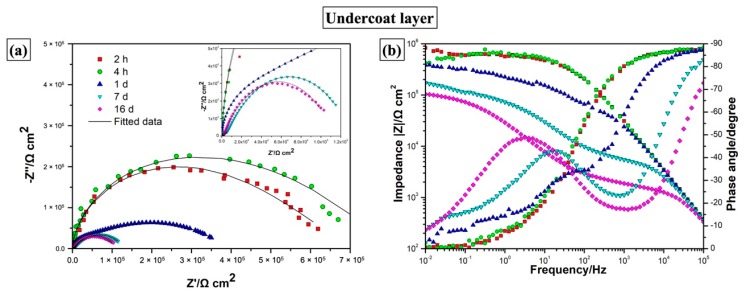
(**a**) Nyquist plots and (**b**) Bode plots obtained from EIS measurements for the one-layer coating immersed in 5% NaCl after different immersion times (2 h, 4 h, 1 d, 7 d, 16 d). The solid lines show the fitted experimental data.

Comparative electrochemical measurements were also conducted on the one-layer coating without the topcoat. As presented in the Bode plots in Figure 11, the electrochemical properties of the coating layer remained almost unchanged after 4 h of immersion. However, the barrier properties of the coating film significantly deteriorated with prolonged immersion time, as indicated by the sharply decreased impedance values in the Bode plots and shrunk impedance arcs in the Nyquist plots. The fast coating deterioration is also confirmed by the visual appearance of the coating surface. As presented in photograph in Figure 10b, the underlying layer without the topcoat was heavily corroded and covered with rust just after 16 d immersion in NaCl solution. For the one-layer coating, two time constants were also distinguishable in the phase angle diagrams after 2 h immersion in chloride solution. We attribute the high-frequency and low-frequency time constants to the coating film and coating/metal interface, respectively, as the corrosion reactions have been visually observed on the metallic substrates.

In comparison with the EIS results from the two-layer structure, the |Z|_0.01Hz_ values of the one-layer coating at the early phase of immersion were almost three orders of magnitude lower, and the Nyquist plots also exhibited smaller impedance arcs comparing to these of two-layer coating, implicating poorer anticorrosion performance. The comparison of electrochemical behaviors of two coating structures suggests that the application of the thin topcoat resulted in a significantly better barrier and anticorrosion performance, as well as longer durability of protective function.

**Figure 12:**
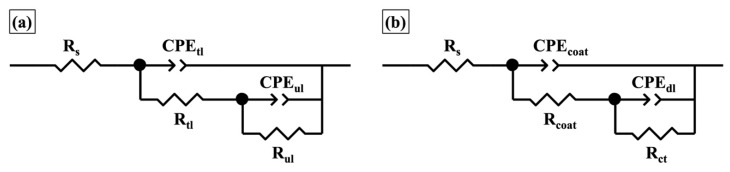
Schemes of equivalent circuits used to fit EIS data for (**a**) two-layer coating, and (**b**) one-layer coating without the topcoat.

To further elaborate on the electrochemical properties of both coating structures, the EIS spectra after different immersion times were fitted using the equivalent circuits depicted in Figure 12, which are the widely used equivalent circuit designs in the literature [5,38,42,43,44]. Here, constant phase elements (CPEs) instead of ideal capacitors were used to better fit the experimental results, and the impedance of a CPE is defined by *Z*_CPE_ = *Y*_0_^−1^*(jw)*^−*n*^, where *w* is the angular frequency, and *Y*_0_ and *n* represent the calculated parameters for the CPEs [42]. The equivalent circuit in Figure 12a was used to fit EIS data for the two-layer coating, where *R*_s_ represents the solution resistance, CPE_tl_ and *R*_tl_ are interpreted as the constant phase element and resistance of the top layer, and CPE_ul_ and *R*_ul_ are interpreted as the constant phase element and resistance of the underlying layer. The EIS data for the one-layer coating was fitted using the equivalent circuit in Figure 12b, where CPE_coat_ and *R*_coat_ correspond to the constant phase element and resistance of sol-gel film, and CPE_dl_ and *R*_ct_ represent the double layer constant phase element and charge transfer resistance at the coating/metal interface.

As shown in Table 2, the *R*_tl_ and *R*_ul_ values for the two-layer structure remained steady over the whole test period, fluctuating around 2 × 10^6^ and 3 × 10^8^ Ω·cm^2^ with elapsed immersion time, respectively. The *R*_ul_ values were two orders of magnitude higher than *R*_tl_ values, possibly due to the higher thickness of the underlying layer. The fitted data for constant phase elements CPE_tl_ and CPE_ul_ also revealed steady values with some fluctuation, indicating that the corrosion prevention capabilities of these two coating layers remained effective, and very few ingresses of water occurred in the coating film.

For the one-layer coating, the *R*_c_ and *R*_ct_ values generally represent the electrolyte penetration capability of the coatings and corrosion resistance behaviors at the coating/metal interface, respectively [44]. As shown in Table 2, both *R*_c_ and *R*_ct_ values dropped drastically with elapsed immersion time, indicative of rapid deterioration of the coating film and its corrosion resistance. Additionally, the *R*_c_ value of one-layer coating decreased more sharply than its *R*_ct_ value, indicating that the corrosion process may have caused more detrimental damage to the coating itself than to the coating/metal interface, possibly resulting from more water uptake in the pore structure of the coating layer. This was also consistent with the discovery in the Bode plots that the |Z| value dropped more evidently in the low-frequency range. On the other hand, CPE_c_ and CPE_dl_ values mostly reflect the water uptake of the coating and water spreading at the coating/metal interface, respectively. The simulated CPE_dl_ values of the one-layer coating increased more rapidly than the CPE_c_ values, which can be ascribed to increased water accumulation at the coating/metal interface after water molecules passed through the pinholes and defects in the underlying layer structure.

Overall, the one-layer coating showed poorer electrochemical properties compared to the two-layer coating structure. The EIS results collectively indicated that the topcoat of the coating structure played a crucial role in limiting the transport of corrosive species. The direct exposure of the underlying layer had a detrimental impact on the integrity of barrier properties of the sol-gel matrix. This may have resulted from the lower hydrophobicity, larger surface area and high porosity of the underlying layer surface, which led to easier access for the electrolyte media to pass through the surface pinholes and pathways in the coating structure and reach the coating/substrate interface.

## 4. Conclusions

In conclusion, a preparation method of sol-gel derived coating system with a two-layer structural design was proposed. The white-colored coating was comprised of inexpensive raw materials, including commercially available MTMS silane crosslinker and colloidal SiO_2_ nanoparticles, together with silica and titania powders as fillers and white pigments. The as-prepared coatings exhibited uniform and defect-free film surfaces with enhanced coating thickness, high hardness and strong adhesion on Q235 carbon steel substrates. Combining the results from the anticorrosion tests, including the salt spray test, outdoor exposure experiment, and EIS measurements, the two-layer coating system exhibited improved long-term corrosion prevention durability. This unique structure maintains the desirable physical properties of ceramic-based sol-gel coatings while exhibiting better barrier function and corrosion prevention performance. Comparative electrochemical studies also revealed that the application of a topcoat could considerably enhance the anticorrosion performance, possibly due to its smoother surface features and increased surface hydrophobicity. Overall, this study provided new insights into a new design paradigm for sol-gel derived coatings with low-cost and facile preparation, and improved anticorrosive properties.

## Figures and Tables

**Table 1:** Elemental compositions of the top and underlying layer of the coatings obtained by energy dispersive spectroscopy (EDS) analysis at the top section and cross-section of the coatings.

Elements	Top Layer (Top View) (at.%)	Top Layer (Cross-Section) (at.%)	Underlying Layer (Top View) (at.%)	Underlying Layer (Cross-Section) (at.%)
O	61.8 ± 4.1	58.7 ± 4.9	62.3 ± 4.4	65.6 ± 6.2
Si	38.1 ± 1.4	41.3 ± 1.8	31.8 ± 1.3	28.8 ± 1.5
Ti	0.1 ± 0.0	0.1 ± 0.0	5.9 ± 0.3	5.6 ± 0.3

**Table 2:** Electrochemical parameters of the two coatings on carbon steel surface obtained after fitting the experimental EIS spectra.

Coating	Immersion Time	*R*_tl_ (Ω·cm^2^)	CPE_tl_		*R*_ul_ (Ω·cm^2^)	CPE_ul_		χ^2^ 10^−3^
			*Y*_0_ (Ω^−1^·s^n^·cm^−2^)	*n*		*Y*_0_ (Ω^−1^·s^n^·cm^−2^)	*n*	
Two-layer	1 d	2.11 ± 0.02 × 10^6^	4.38 ± 0.06 × 10^−9^	0.956 ± 0.001	3.17 ± 0.06 × 10^8^	2.32 ± 0.02 × 10^−8^	0.778 ± 0.003	1.9881
	5 d	1.90 ± 0.04 × 10^6^	4.66 ± 0.07 × 10^−9^	0.952 ± 0.002	2.84 ± 0.05 × 10^8^	2.35 ± 0.02 × 10^−8^	0.786 ± 0.003	1.9358
	26 d	1.94 ± 0.06 × 10^6^	5.21 ± 0.09 × 10^−9^	0.947 ± 0.002	3.18 ± 0.07 × 10^8^	2.27 ± 0.02 × 10^−8^	0.789 ± 0.003	2.0837
	63 d	2.64 ± 0.06 × 10^6^	5.49 ± 0.08 × 10^−9^	0.946 ± 0.001	4.33 ± 0.11 × 10^8^	2.11 ± 0.02 × 10^−8^	0.787 ± 0.004	2.2237
	99 d	1.82 ± 0.04 × 10^6^	6.00 ± 0.09 × 10^−9^	0.941 ± 0.002	2.97 ± 0.06 × 10^8^	2.23 ± 0.02 × 10^−8^	0.786 ± 0.003	1.9318
One-layer	**Immersion Time**	***R*_coat_ (Ω·cm^2^)**	**CPE_coat_**		***R*_ct_ (Ω·cm^2^)**	**CPE_dl_**		**χ^2^ 10^−3^**
	2 h	1.14 ± 0.43 × 10^5^	6.27 ± 0.23 × 10^−9^	0.971 ± 0.003	5.88 ± 0.64 × 10^5^	1.90 ± 0.20 × 10^−7^	0.409 ± 0.030	1.1674
	4 h	2.00 ± 0.44 × 10^5^	6.46 ± 0.25 × 10^−9^	0.969 ± 0.003	6.09 ± 0.75 × 10^5^	1.82 ± 0.29 × 10^−7^	0.476 ± 0.050	2.9885
	1 d	2.21 ± 0.38 × 10^4^	5.40 ± 0.49 × 10^−9^	0.984 ± 0.007	3.70 ± 0.12 × 10^5^	1.05 ± 0.05 × 10^−6^	0.409 ± 0.014	5.7427
	7 d	4.66 ± 0.08 × 10^3^	7.33 ± 0.53 × 10^−9^	0.962 ± 0.006	1.27 ± 0.02 × 10^5^	2.64 ± 0.06 × 10^−6^	0.620 ± 0.005	2.2603
	16 d	1.71 ± 0.02 × 10^3^	2.49 ± 0.24 × 10^−8^	0.872 ± 0.008	1.11 ± 0.01 × 10^5^	9.50 ± 0.12 × 10^−6^	0.647 ± 0.003	2.2467
